# Supplementary material for: Waveguide-PAINT offers an open platform for large field-of-view super-resolution imaging
Source: Nat Commun. 2019 Mar 20;10:1267. doi: 10.1038/s41467-019-09247-1 (PMC6427008; doi:10.1038/s41467-019-09247-1)
Supplement: Supplementary file 1 — Supplementary Information [file 41467_2019_9247_MOESM1_ESM.pdf]

Waveguide-PAINT offers an open platform for large field-of-view super-resolution imaging

Archetti et al.

Supplementary Information

Contains Supplementary Figures 1-18, Supplementary Table 1-2, Supplementary Notes 1-3 and Supplementary References

SUPPLEMENTARY FIGURES

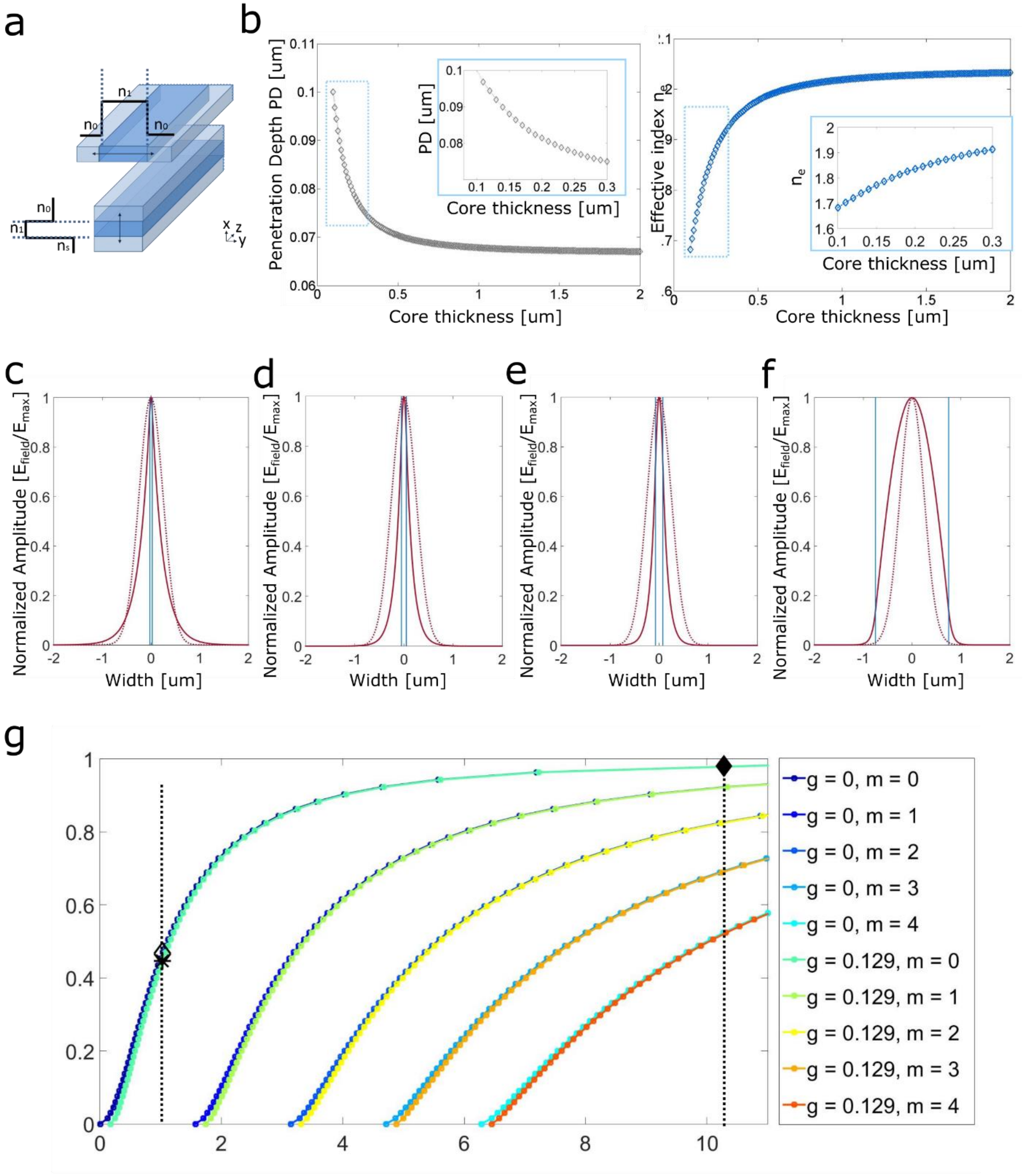

Supplementary Figure 1 1D TE0 waveguide mode simulations. The simulations were performed using a slab-waveguide approximation in *X* and *Y* directions (**a**). In the *X* direction the top cladding corresponds to the sample media with a refractive index of n_0 = 1.38. In the *Y* direction the lateral cladding is SiO_2 with a

refractive index of n_0 = 1.47. All simulations were performed with a light wavelength of 647 nm and with a waveguide core with a refractive index of n_1 = 2.04. **b,** Penetration depth simulation as a function of the waveguide core thickness shows that the expected penetration depth is roughly 90nm for a 150 nm Si_3N_4 core waveguide excited with 647 nm light wavelength. Grey and blue diamond symbols represent the simulated penetration depth and effective index respectively. Simulations **c-f** show that decreasing (from **f** to **c**) the input taper width leads to a better overlap between fundamental mode excitation and beam field. Red dashed and continuous lines correspond to the simulated beam input and mode profile respectively. Blue vertical lines correspond to the waveguide core-cladding interface positions. **c,** Taper input width 50nm. **d,** Taper input width 100 nm. **e,** Taper input width 200 nm. **f,** Taper input width 1.5 µm. **g,** The simulated dispersion curves for both the two direction *X* (with a asymmetry coefficient gamma = 0.1287) and *Y* (gamma = 0), show that for the both directions (black start indicates the result for the *X* direction where the core width is 150 nm, *v* = 1.028, *b* = 0.447, *n*e = 1.74; black-no fill-diamond indicate the result for the *Y* direction where the input taper width is 150nm, *v* = 1.028, *b* = 0.466 and *n*e = 1,76) the single mode excitation is ensured. While for *Y* direction where the input taper width is 1500nm (black fill diamond), *v* = 1.028, *b* = 0.466 and *n*e = 1,76) the single mode excitation is not ensured. The input beam size is estimated based on [1].

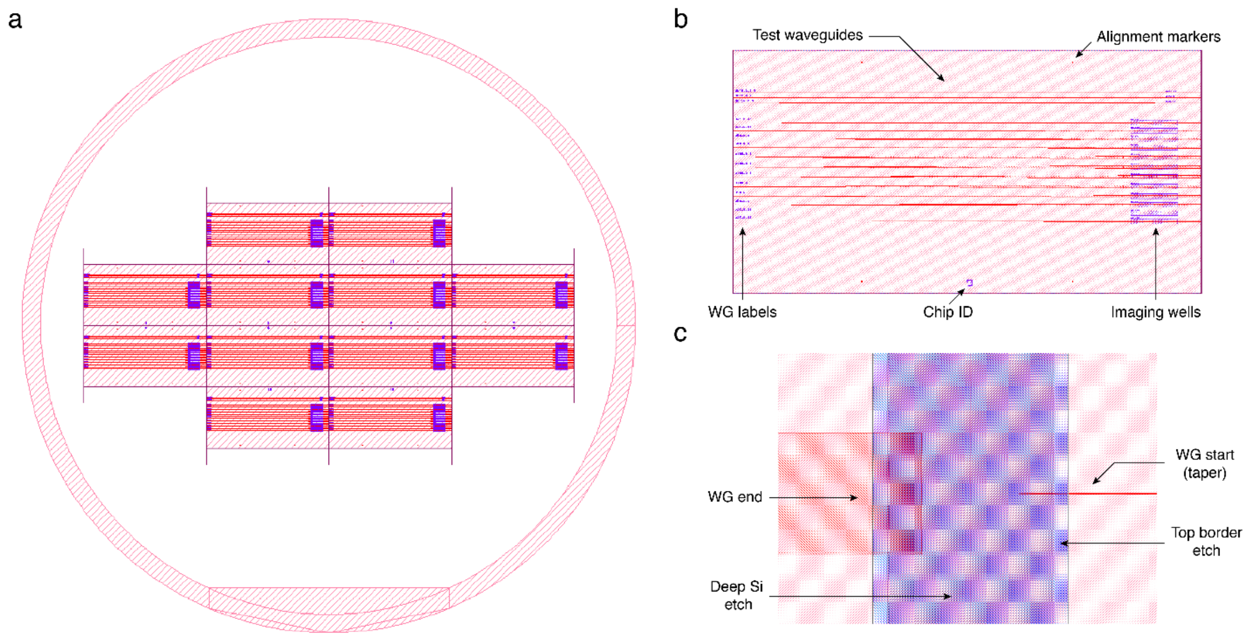

Supplementary Figure 2 Layout of the waveguide chips for cleanroom fabrication (4 functional layers). **a**, 12 chips, each containing 12 imaging waveguides, on the standard 4 inch (100 mm) wafer. **b**, Closer look at one of twelve chips showing additional design features such as the imaging wells (on the right side), the waveguide labels at the input nanotaper tips and the chip identification number (ID). **c**, Zoom-in on the border between two chips and the etching areas for the two-step lithography and etching process.

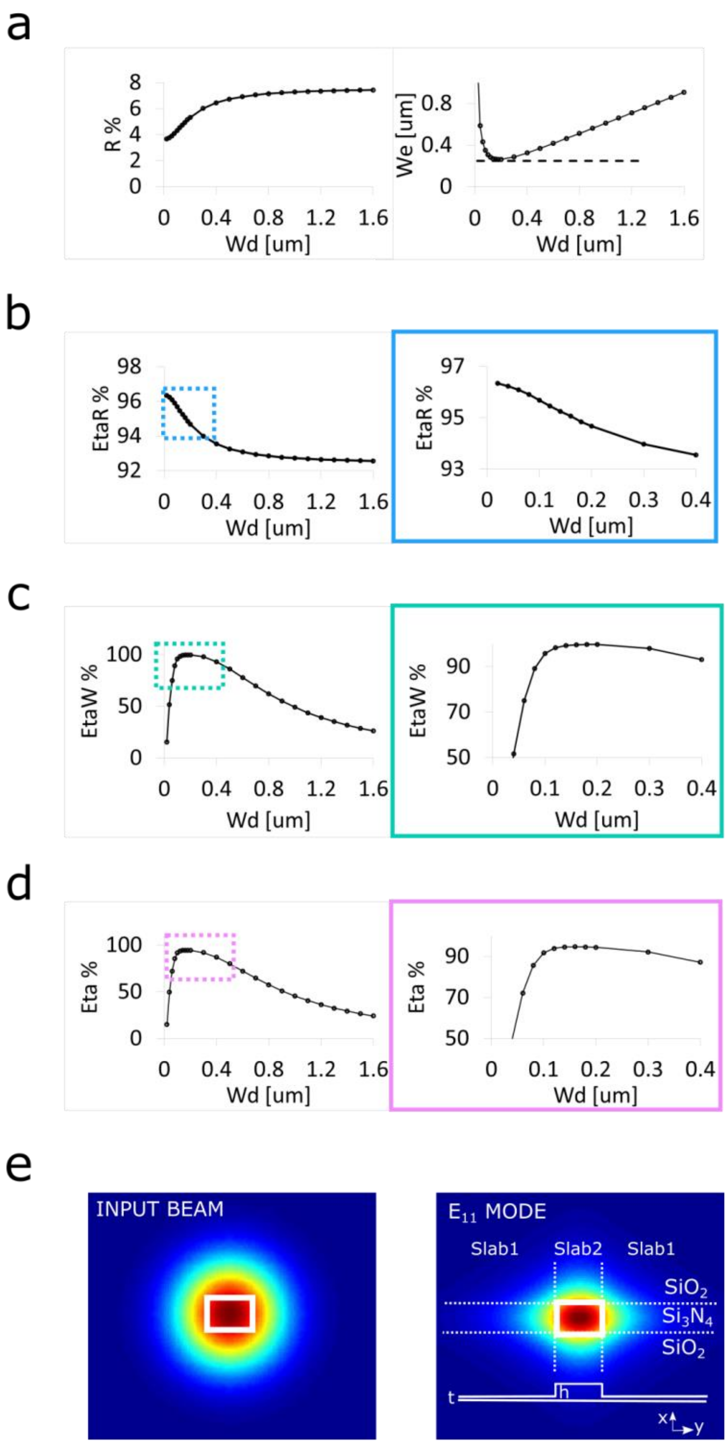

Supplementary Figure 3 Coupling efficiency and E11 waveguide mode profile at the input taper tip. A 150 nm thick Si_3N_4 waveguide core (refractive index of n_1 = 2.038 at 647 nm) is surrounded by SiO_2 (refractive index n_0 = 1.47 at 647 nm). **a,** The two main contributions to coupling losses are due to Fresnel reflection (Left) and the waveguide field mode and input beam field distribution mismatch (Right). **b,** Transmission

efficiency η_R. **c,** Field distribution efficiency, η_W. **d**, The final coupling efficiency η is the product of the transmission (η_R) and mode overlap efficiency (η_W). The effective index of the waveguide (fundamental to compute the main waveguide properties such as the effective mode width and the reflection) has been computed using the effective index approximation method (**e**).

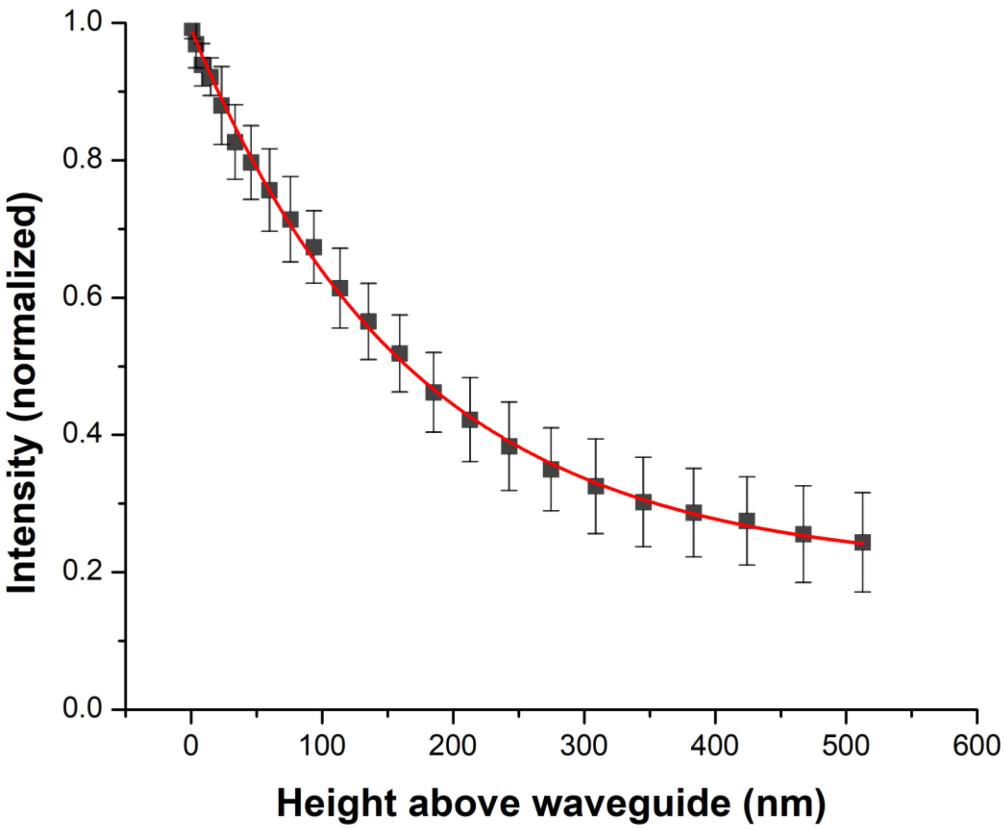

Supplementary Figure 4 Penetration Depth measurements were performed using beads with a mean diameter of 6.5 µm labelled with Atto647. The penetration depth can be extracted measuring the individual bead intensity profiles[2]. Approximating the bead as a sphere, and expressing the height above the waveguide (*d*) as a function of the local radius (r_d), the penetration depth can be extracted as the decay length of an exponential distribution of the normalized intensity as a function of *d*. We found the average penetration depth to be 94 ± 24 nm. Error bars correspond to the standard deviation of the beads measurements.

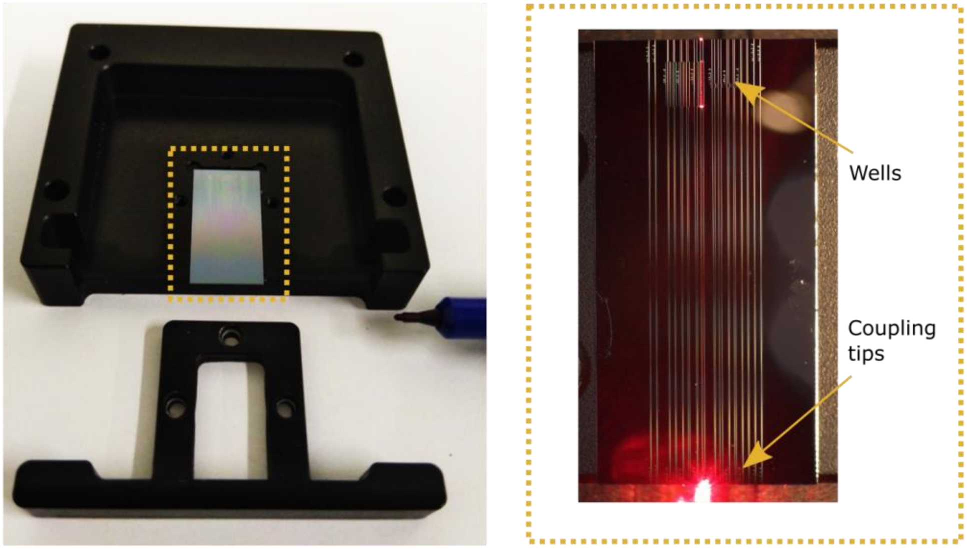

Supplementary Figure 5 Waveguide chip and chip holder integration. The chip lies on its holder positioning slot (Left). On the right, the magnified view of the chip shows that an efficient coupling is established when the light scattered from the well is maximized. The image has been recorded with Digital Microscope 1.3 MPixel, AM4815ZT, Dino-Lite. The total length of the chip is 2 cm.

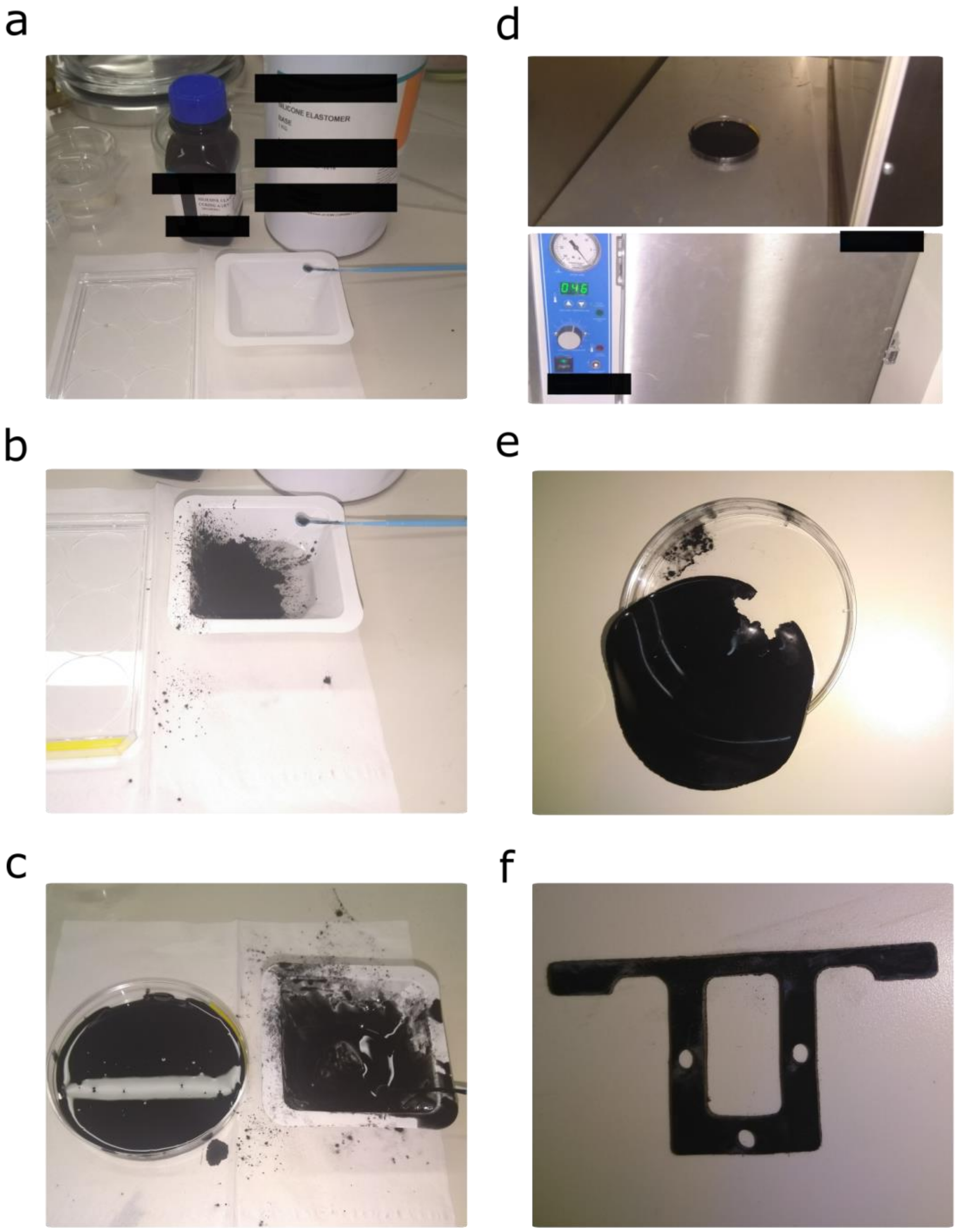

Supplementary Figure 6 PDMS strip creation. **a,** 2% of curing agent is mixed with the Silicone Elastomer base. **b,** The black toner powder is added till the silicone mixture is not transparent anymore. **c**, The viscous mixture is poured on a flat surface such as the surface of a cell plate. **d**, The plate is placed in an oven at 50°C for 4h. **e**, The PDMS layer is easily detached from the plate using a spoon flat back and it is ready for laser cut. **f,** PDMS strip fits the holder-gate trench to prevent fluid leakage out (see Supplementary Fig. 5).

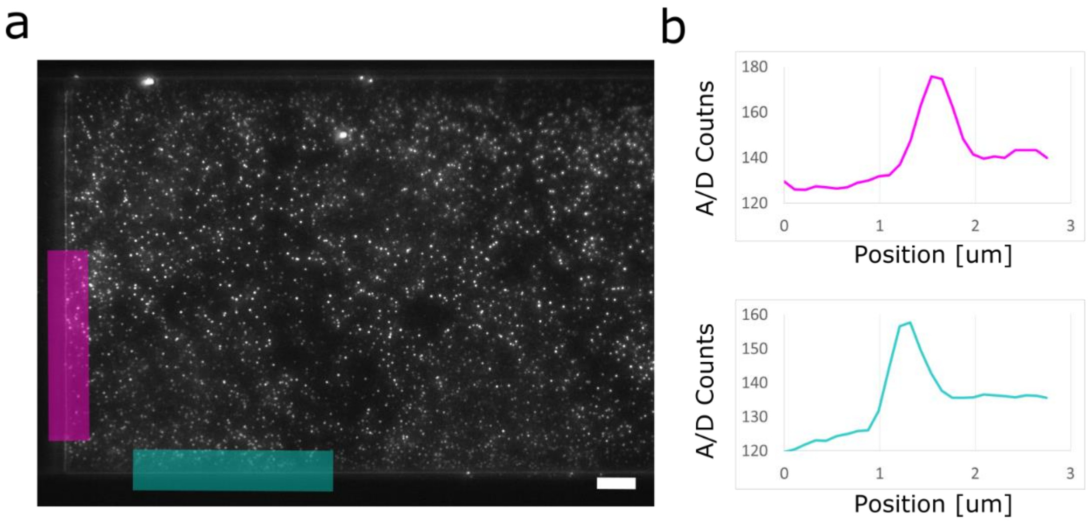

Supplementary Figure 7 Light scattering at the edges of the waveguide well. a, Single raw image of a typical field of view of the waveguide well including the well edges. **b,** The intensity profiles across the well edges show that the scattered light generated at the interface between top cladding and sample media drop in about 1um in both vertical (magenta) and horizontal (cyan) directions. Scale bar 10 µm (**a**).

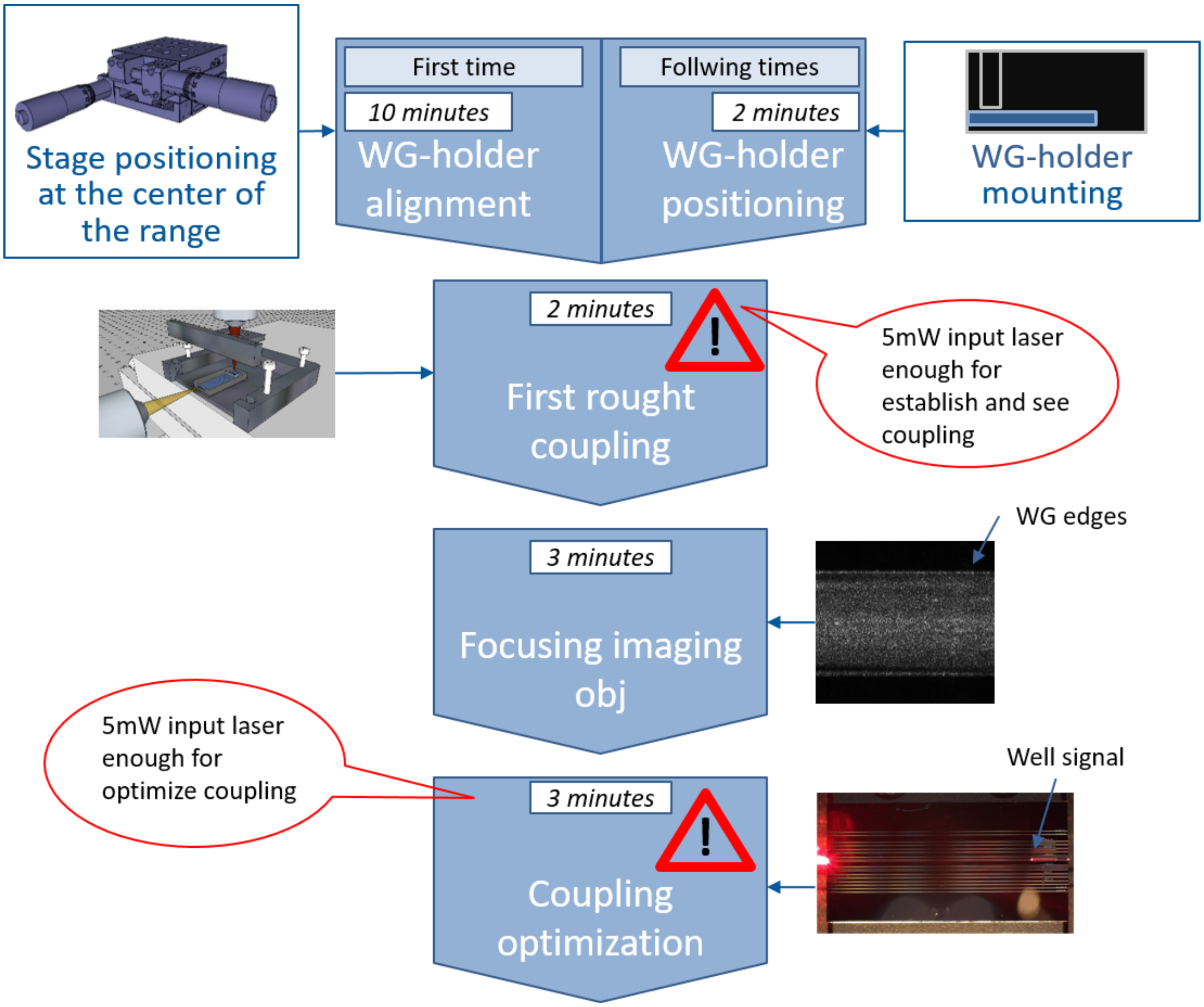

Supplementary Figure 8 Workflow for the first use of the waveguide chip holder and chip in the proposed upright microscope. For the first chip-holder and microscope alignment, position all the stages at the center of their range before starting the coupling procedure. Step 1) Align the waveguide holder at the center of the vertical imaging column. This step requires approximately 10' the first time the holder-and horizontal stage system is aligned with the imaging column. The following times, the holder can be screwed directly on the stage without further alignment. Step 2) Use the three axis stage for a first rough coupling. Step 3) Remove any excitation filter to find the focus. Use the waveguide edges as reference. Step 4) Optimize the coupling using the spackle pattern generated by the chip input facet to define the focus position.

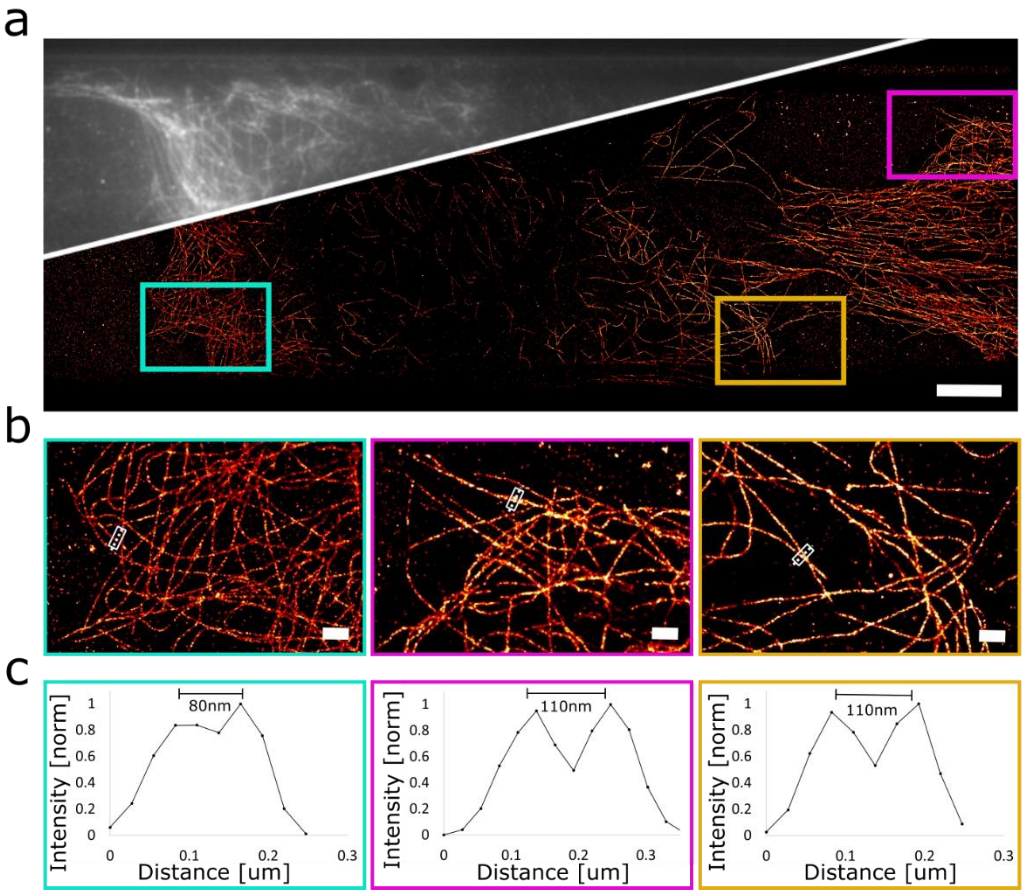

Supplementary Figure 9 Diffraction-limited and DNA-PAINT imaging of microtubules in COS7 cells. **a,** Single field of view DNA-PAINT reconstruction of COS7 cells labeled with antibodies against α-tubulin and imaged using 500 pM imager strand (I1-560, Ultivue Duplex Kit). Green channel. **b,** Magnified views of the boxed regions show microtubules well-resolved across the field of view. **c**, Intensity profiles across two microtubules reveal two peaks. Scale bar 20 µm (**a**), 1 µm (**b**).

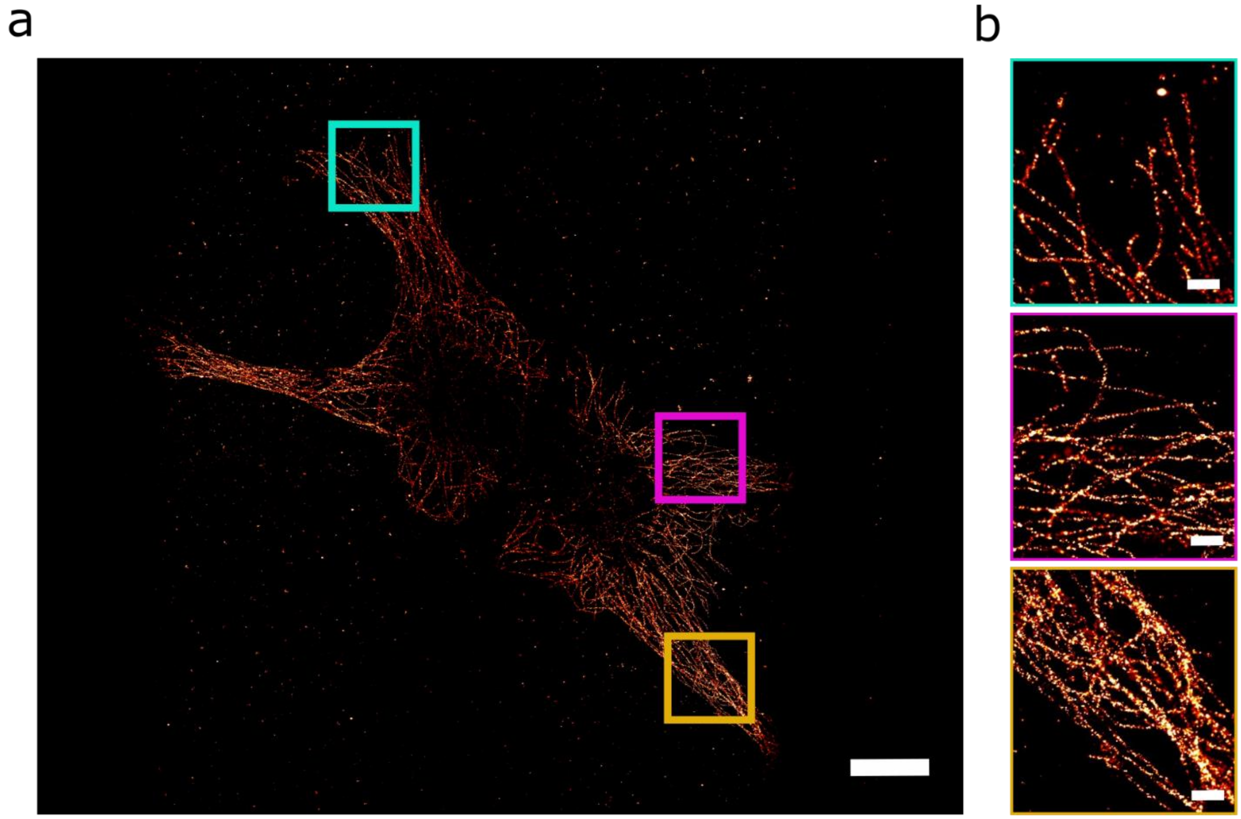

Supplementary Figure 10 DNA-PAINT imaging of microtubules in COS7 cells. **a,** Single field of view DNA-PAINT reconstruction of COS7 cells labeled with antibodies against α-tubulin and imaged using 500 pM imager strand (I1-655, Ultivue Duplex Kit). Red channel. **b,** Magnified views of the boxed regions show microtubules well-resolved across the field of view. Scale bar 10 µm (**a**), 1 µm (**b**).

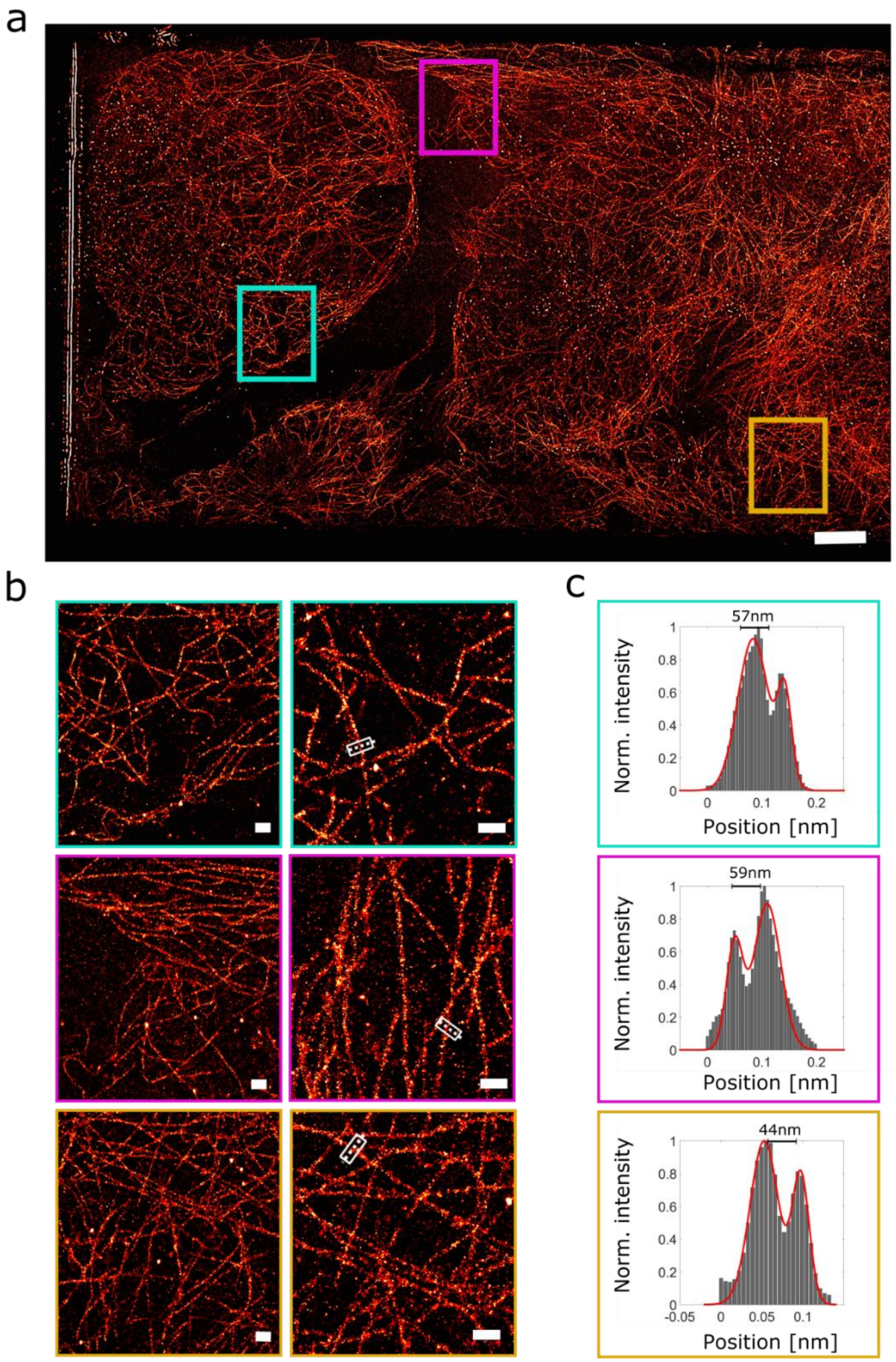

Supplementary Figure 11 Waveguide-PAINT imaging of microtubules in COS7 cells. **a,** Single field of view DNA-PAINT reconstruction of COS7 cells labeled with antibodies against α-tubulin and imaged using 500 pM imager strand (I1-655, Ultivue Duplex Kit) (left panel). **b**, Magnified views of the boxed regions (left panel, from top to bottom) show microtubules well-resolved across the field of view (a, right panel). **c**,

Intensity profiles across individual microtubules (a, right panel) reveal two peaks that can be described with the sum of two Gaussian functions (**b**, red line). Scale bar 10 µm (**a**) and 1 µm (**b**).

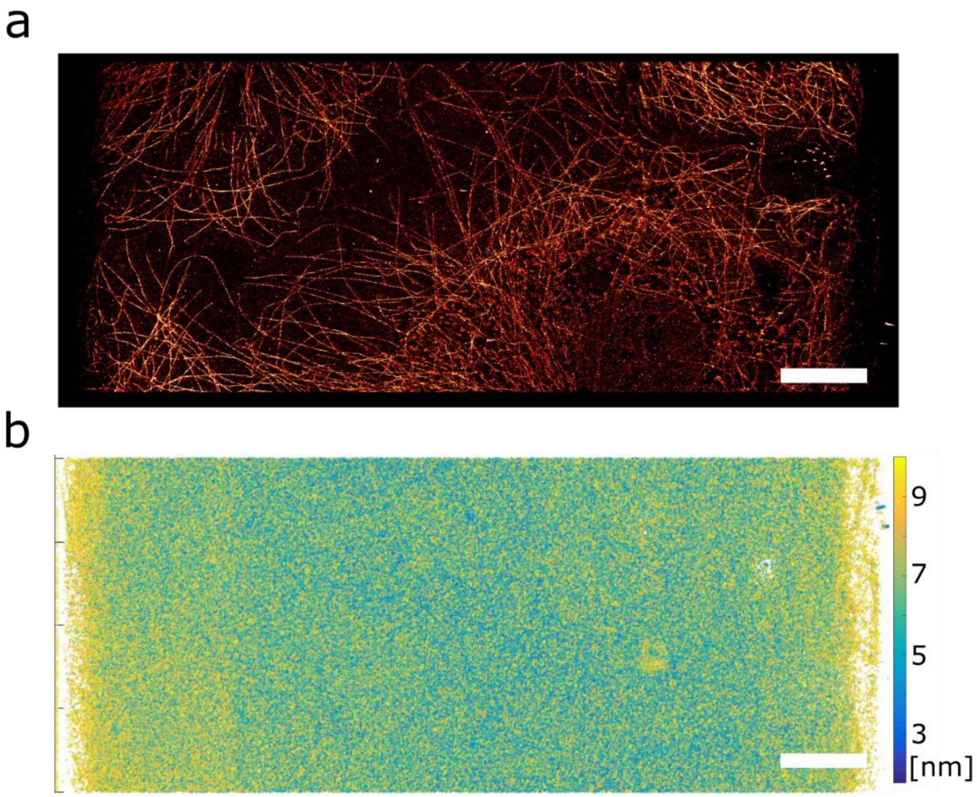

Supplementary Figure 12 Localization precision across the waveguide width. **a,** Superresolved image section across the waveguide. **b,** Corresponding scatter-plot of the localizations with color-map proportional to the localization precision. The localization precision is computed using [3] which represents the best case scenario for the actual localization error. Scale bar 10 µm.

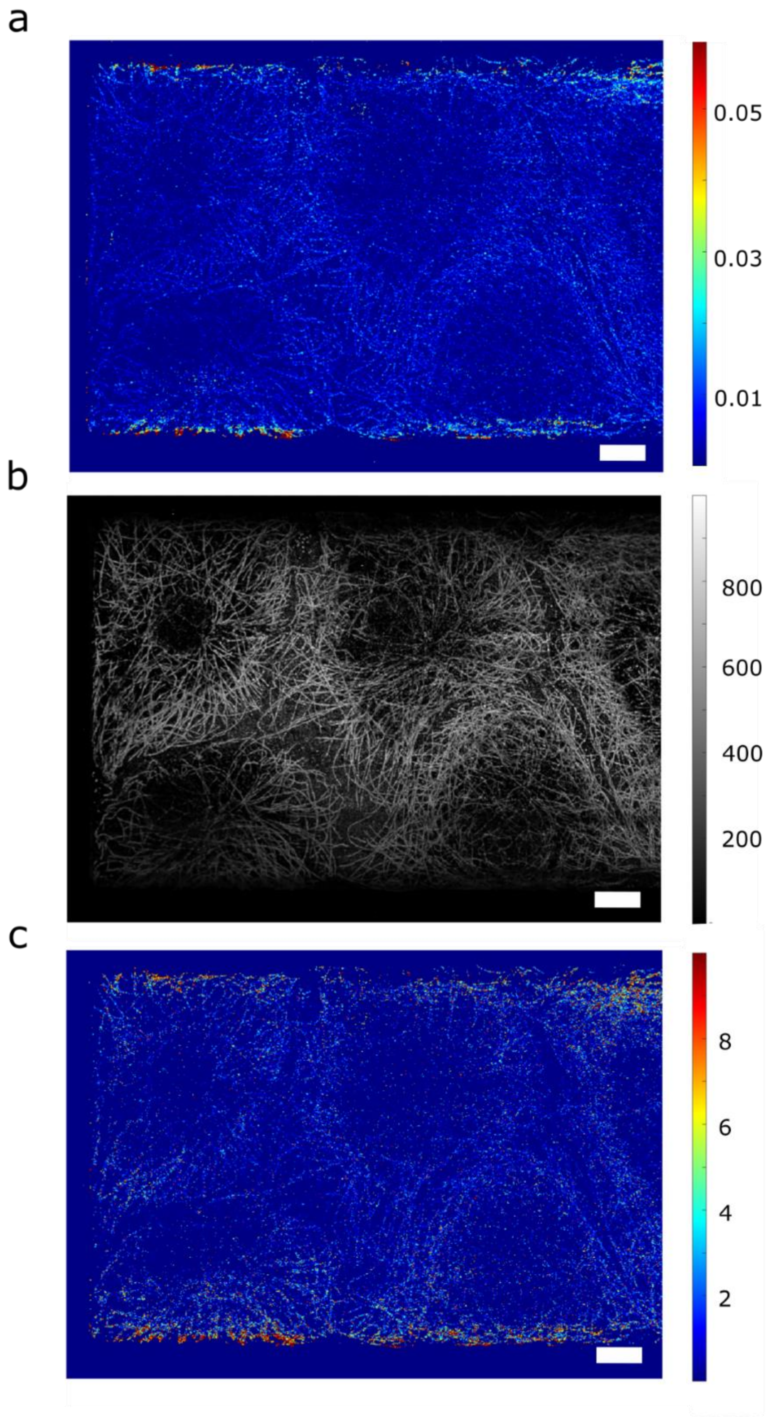

Supplementary Figure 13 DNA-PAINT raw data reconstructed and analyzed using the super-resolution optical fluctuation imaging SOFI[4] approach. **a,** The estimated on-time ratio values as well as molecular brightness (**b**) and density (**c**) exhibit a nearly homogeneous distribution over the whole field of view except for the areas near the edges of the waveguide (due to increased scattering there). Background of the input

image sequence undergoes periodic fluctuations with period approximatively equal to 25 frames corresponding to the rotation period of the rotating polarizer. The majority of emitters fluctuate with a period approximatively equal to 172 frames (corresponding to 26 s; on-time ratio 0.0058). Due to the large data size, the acquired image series were processed in 4 separate sequences (4, 4, 4, and 5 files each). Drift was estimated using normalized phase cross correlation method. Each input image was drift corrected with subpixel precision by bilinear interpolation and then processed by SOFI algorithm. Results from 4 separate sequences were mutually co-registered and then averaged to get the final SOFI images and estimation of molecular parameters (brightness, density, on-time ratio). Scale bar 10 µm.

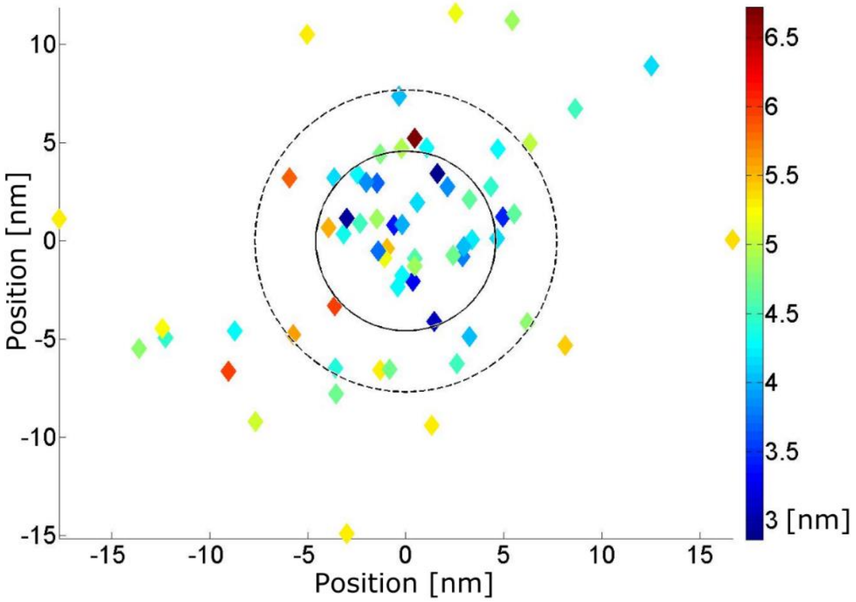

Supplementary Figure 14 Comparison between theoretical and measured localization precision. We collected the data from 12 origami sites and aligned their centers of mass. This allows us to collect statistics on 64 molecules.

Diamond points represent the measured positions of the molecules visiting the origami sites. The color scale encodes the localization precision of each molecule calculated theoretically[3]. The black continuous circle represents the average localization precision calculated theoretically[3] (radius equal to 4.6 ± 0.7 nm) while the dashed circle represents the standard deviation of the molecule positions (radius equal to 7.7 nm).

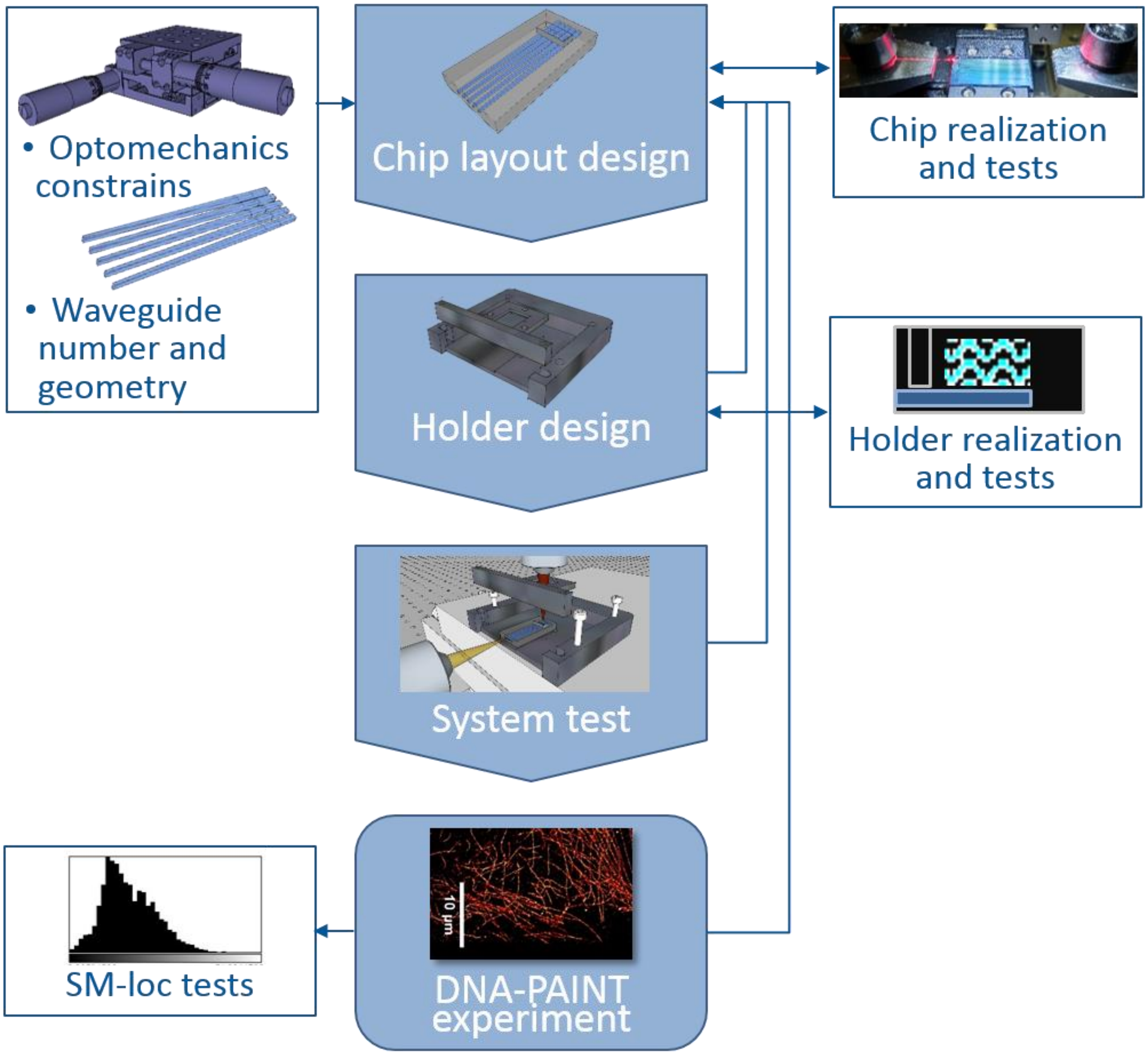

Supplementary Figure 15 Waveguide-PAINT platform realization workflow. After having evaluated the optomechanics constraints of the microscope, the user can define the chip geometry that better suits the experiments. Once the chip geometrical parameters are designed, the user can modify the Waveguide-PAINT platform design to adapt it to the desired experimental conditions. Before starting the DNA-PAINT experiments the system needs to be tested in order to check both the chip coupling and buffer holding. Finally, the user can perform his first DNA-PAINT experiment to test whether the desired single molecule localization (SM-loc) precision is achieved.

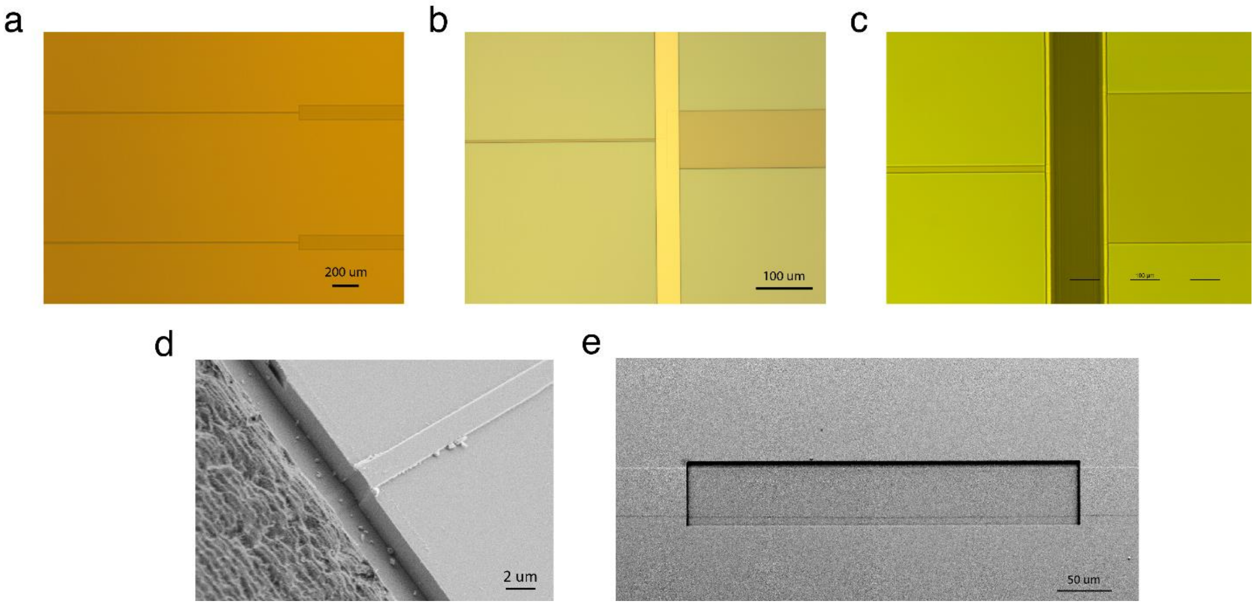

Supplementary Figure 16 Inspections of the waveguides at intermediate steps during the fabrication process. **a**, After etching the waveguides. **b**, After etching the wells and top chip borders. **c**, After deep etching of silicon substrate. **d**, SEM image of the chip border after 2-step lithography and etching process to protect the input facet. **e**, Imaging well etched in the top SiO_2 cladding.

a

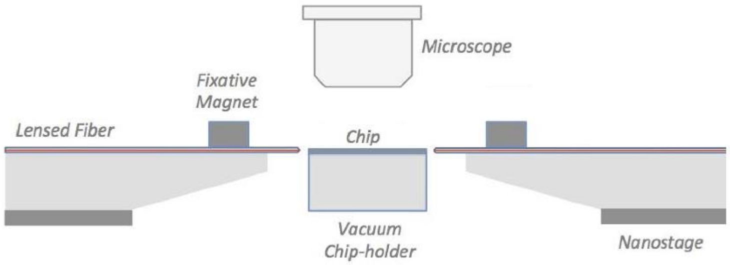

b

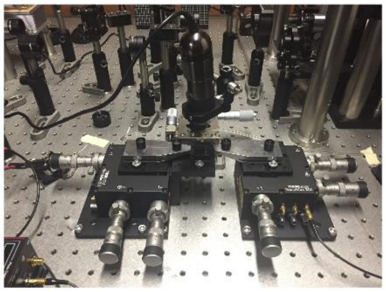

c

	No taper	Linear	Quadratic	Parabolic
**Mode profile after the taper**	highly multimode	Single-mode	Perturbed single-mode with side peaks	Perturbed single-mode with side peaks
**Transmission (average)**	< 5%	35%	25%	5%

Supplementary Figure 17 Chip characterization and transmission measurements of various expansion tapers. **a**, Sketch of the simple setup, built to perform the transmission measurements. **b**, Photograph of the assembled setup, consisting of the following parts: 2xThorlabs MAX311D/M nanopositioners with MMP1 top plates , 2xThorlabs HFV002 holders, 1xThorlabs AMA009 extension platform, 1xThorlabs HWV001 vacuum holder, 2xOZ Optics lensed fibers, 1xADAFCB4 connector, 1xThorlabs PM100A power meter and 1xDyno-Lite USB-microscope to check the alignment. **c**, Results of the transmission measurements (averaged over multiple chips and waveguides) for different types of tapers and the simulated mode profiles at the end of the waveguide.

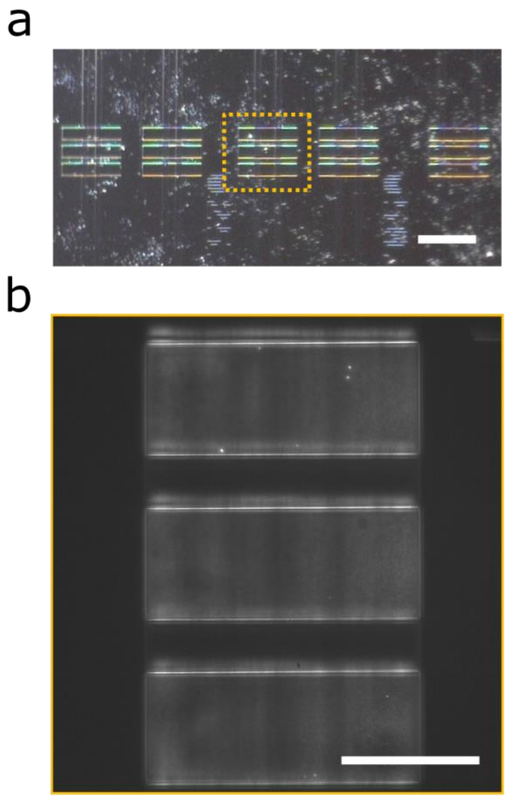

Supplementary Figure 18 Multi-well waveguide chip fabrication. The presence of 3 wells within the same field of view provide a high-throughput platform for screening of different sample conditions. **a,** Low magnification imaging of multiple waveguides with multiple wells. **b**, Multiple wells imaged with the waveguide chip and chip holder setup. Scale bar 2 mm (**a**) and 50 µm (**b**).

SUPPLEMENTARY TABLES

Fabrication process

Step	Process description
01	Si3N4 150 nm high-stress LPCVD deposition (Waveguide core layer)
02	E-beam lithography (Waveguides & alignment markers)<br>(ZEP, 300 nm; dose: 180 uC/cm^2 @ 100 kV)
03	Si_3N_4 RIE (waveguide realization)<br>(SPTS APS, CHF_3/SF_6 chemistry; etch time: 60 s)
04	SiO_2 2 µm LPCVD deposition (top cladding layer)<br>(Centrotherm furnace, Low Temperature Oxide, SiH4 @ 450°C)
05	Photolithography (Imaging well & top borders)<br>(Heidelberg MLA150; AZ ECI, 1.5 µm; dose: 170 mJ/cm^2 @ 405 nm)
06	SiO_2 RIE (Imaging well and top cladding border etching)<br>(SPTS APS, He/C_4F_8 chemistry; etch time: 4 min 51 s)
07	Photolithography (Borders & top facets)<br>(Heidelberg MLA150; AZ ECI, 1.5 µm; dose: 170 mJ/cm^2 @ 405 nm)
08	SiO_2/ Si_3N_4 RIE (Core and bottom cladding etching)<br>(SPTS APS, He/C_4F_8 chemistry; etch time: 10 min)
09	Photolithography (Chip borders for deep etching)<br>(Heidelberg MLA150; AZ 9260, 5.5 µm; dose: 255 mJ/cm^2 @ 405 nm)
10	Si deep-etching (Deep etching of chip borders)<br>(Adixen AMS200, Bosch process, etch time: 75 min; etch depth: 300 µm)
11	Si backside grinding (Splitting the chips)<br>(DAG810 automatic surface grinder)

Supplementary Table 1 Chip fabrication process: main steps

Experimental condition and localization results

Sample/ Figure	**Exposure time [ms]**	**Imager concentration [nM]**	**Laser λ [nm]**	**Laser power [mW]**	**Number of Frames**	**Loc. precision [nm]**	**Photons per loc.**	**Analysis Algorithm**
Fig.3a	150	0.5	647	100	25000	6± 2	6250±3770	Spline, with experimental PSF[5], and TunderStorm
Fig.3c	300	0.5	647	100	10000	4 ± 1	9420± 5600	LS, multi-emitter, ThunderStorm[6]
Supp. Fig. 10	100	0.5	565	55	10000	13 ± 5	2050 ± 1500	LS, multi-emitter, ThunderStorm[6]
Supp. Fig. 11	50	0.2	641	100	10000	16 ± 6	1760 ±1250	LS, multi-emitter, ThunderStorm[6]
Supp. Fig. 12	100	0.5	641	100	20000	13 ± 5	2770 ± 2300	LS, multi-emitter, ThunderStorm[6]
Supp. Fig. 14		See Fig. 3a						SOFI

Supplementary Table 2 Experimental condition and localization results

SUPPLEMENTARY NOTES

Supplementary Note 1: Penetration depth and TE0 waveguide mode study

The main waveguide parameters were investigated solving Maxwell's equations with a slab-waveguide approximation in both *X* and *Y* direction (Supplementary Fig. 1b bottom and top configuration respectively). Although such approach can not accurately estimate the mode profile, in particular at the waveguide corners, this method provides a set of important parameters fundamental to address basic questions such as: what is the penetration depth in the *X* direction at the well position where the waveguide is 100 um wide (where a slab approximation is more suitable due to the high waveguide thickness-wideness aspect ratio)? Which is the order of magnitude of taper tip width that could provide a better overlap with the input beam compared to the 1.5 µm proposed in [7]? Could the input taper tip width provide a single mode excitation?

All the simulations were performed with a light wavelength of 647 nm and with a waveguide core with a refractive index of n_1 = 2.04 corresponding to the Si_3N_4 at that wavelength. The slab-waveguide approximation in the *X* direction (Supplementary Fig. 1b top) is simulated with a top cladding corresponding to a sample media with a refractive index of n_0 = 1.38. In the *Y* direction (Supplementary Fig. 1 bottom), the lateral cladding is SiO_2 with a refractive index of n_0 = 1.47. All In the first configuration (bottom) the refractive index of top (n_0) and bottom (n_s) cladding are n_0 = 1.38 (sample media refractive index) and n_s = 1.47 (SiO_2). In the second configuration (top) the refractive index of top and bottom cladding are both $n_0 = n_s$ = 1.47 (SiO_2).

Here we define some parameters that will be used in the following analysis:

- n_1: is the refractive index of the waveguide core

- n_s: is the refractive index of the bottom cladding of the waveguide
- n_0: is the refractive index of the top cladding
- k: is the modulus of the component of propagation vector **K** along the direction X perpendicular to the propagation direction Z (see Supplementary Fig. 1b)
- β: is the modulus of the component of propagation vector K along the propagation direction Z
- n_e: is the effective index $n_e = \beta / k$
- v: is the normalized frequency $v = k^2 \times a^2 (n_1^2 - n_s^2)$
- b: is the normalized propagation constant $b = (n_e^2 - n_s^2)/(n_1^2 - n_s^2)$
- $2a$: is the width of the core of the waveguide [µm]
- m: mode numbers
- γ: measure of the waveguide asymmetry $\gamma = (n_s^2 - n_0^2)/(n_1^2 - n_s^2)$

In this analysis we consider only dielectric optical layers (such as SiO_2 and Si_3N_4). Thus, we can set in the Maxwell's equations $\mu_r = 1$, $\varepsilon_r = {n_i}^2$ as well as $\mathbf{M} = 0$, $\mathbf{j} = 0$, where n_i is the refractive index of the waveguide layers ($n_i = n_0$ is the refractive index of the top cladding, $n_i = n_s$ of the substrate, $n_i = n_1$ of the core).

We consider the step-index planar waveguide configuration – called the slab waveguide – shown in Supplementary Fig. 1a.

We choose the direction of light propagation to be along the z-axis and the electromagnetic fields $\mathbf{E}$ and $\mathbf{H}$ without any dependence on the Y axis ($\partial\mathbf{E}/\partial \mathrm{y} = 0$ and $\partial\mathbf{H}/\partial \mathrm{y} = 0$). Thus, Maxwell's equations become

$$\begin{cases} \nabla \times \mathbf{E} = -\frac{\partial \mathbf{B}}{\partial t} = -\mu_0 \frac{\partial (\mathbf{H} + \mathbf{M})}{\partial t} = -\mu_0 \frac{\partial \mathbf{H}}{\partial t} \\ \nabla \times \mathbf{H} = \mathbf{j} + \frac{\partial \mathbf{D}}{\partial t} = \frac{\partial (\varepsilon_0 \boldsymbol{\varepsilon_r} \mathbf{E})}{\partial t} = \varepsilon_0 {n_i}^2 \frac{\partial \mathbf{E}}{\partial t} \end{cases}$$

where

$$\begin{cases} \mathbf{E}(r,t) = \mathbf{E_0}(x,y) e^{\mathrm{j}(\omega t - \boldsymbol{\beta} z)} \\ \mathbf{H}(r,t) = \mathbf{H_0}(x,y) e^{\mathrm{j}(\omega t - \boldsymbol{\beta} z)} \end{cases}$$

$$\begin{cases} \frac{\partial E_z}{\partial y} + \mathrm{j}\beta E_y = -\mathrm{j}\omega\mu_0 H_x \\ -\mathrm{j}\beta E_x - \frac{\partial E_z}{\partial x} = -\mathrm{j}\omega\mu_0 H_y \\ \frac{\partial E_y}{\partial x} - \frac{\partial E_x}{\partial y} = -\mathrm{j}\omega\mu_0 H_z \end{cases} \qquad \begin{cases} \frac{\partial H_z}{\partial y} + \mathrm{j}\beta H_y = \mathrm{j}\omega\varepsilon_0 {n_i}^2 E_x \\ -\mathrm{j}\beta H_x - \frac{\partial H_z}{\partial x} = \mathrm{j}\omega\varepsilon_0 {n_i}^2 E_y \\ \frac{\partial H_y}{\partial x} - \frac{\partial H_x}{\partial y} = \mathrm{j}\omega\varepsilon_0 {n_i}^2 E_z \end{cases}$$

$$\begin{cases} 0 + \mathrm{j}\beta E_y = -\mathrm{j}\omega\mu_0 H_x \\ -\mathrm{j}\beta E_x - 0 = -\mathrm{j}\omega\mu_0 H_y \\ \frac{\partial E_y}{\partial x} - 0 = -\mathrm{j}\omega\mu_0 H_z \end{cases} \qquad \begin{cases} 0 + \mathrm{j}\beta H_y = \mathrm{j}\omega\varepsilon_0 {n_i}^2 E_x \\ -\mathrm{j}\beta H_x - \frac{\partial H_z}{\partial x} = \mathrm{j}\omega\varepsilon_0 {n_i}^2 E_y \\ \frac{\partial H_y}{\partial x} - 0 = \mathrm{j}\omega\varepsilon_0 {n_i}^2 E_z \end{cases}$$

$\Leftrightarrow E_x = H_y = 0$ and $E_z = 0$, thus

$$\begin{cases} -\frac{\beta}{\mu_0\omega} E_y = H_x \\ \frac{1}{j\mu_0\omega}\frac{\partial E_y}{\partial x} = H_z \end{cases} \quad (1); \qquad \frac{\partial H_z}{\partial x} - \mathrm{j}\beta H_x = -\mathrm{j}\omega\varepsilon_0 {n_i}^2 E_y$$

$$\frac{\partial}{\partial x}\left(\frac{1}{j\mu_0\omega}\frac{\partial E_y}{\partial x}\right) + \left(-\frac{j\beta^2}{\mu_0\omega} E_y\right) = -\mathrm{j}\omega\varepsilon_0 {n_i}^2 E_y$$

$$\frac{\partial^2 E_y}{\partial x^2} - \beta^2 E_y = -\omega^2 \varepsilon_0 \mu_0 {n_i}^2 E_y \quad ; \qquad \begin{gathered} \sqrt{\varepsilon_0\mu_0} = \frac{1}{c_0} \\ \boldsymbol{k_0} n = \boldsymbol{k} \\ {k_0}^2 {c_0}^2 = \omega^2 \end{gathered}$$

$$\frac{\partial^2 E_y}{\partial x^2} - \beta^2 E_x = -{k_0}^2 {n_i}^2 E_y$$

$$\frac{\partial^2 E_y}{\partial x^2} + \left({k_0}^2 {n_i}^2 - \beta^2\right) E_y = 0$$

$$\begin{cases} \dfrac{\partial^2 E_y}{\partial x^2} = \left(\beta^2 - {k_0}^2 {n_0}^2\right) E_y & (\mathrm{x} > \mathrm{a}) \\ \dfrac{\partial^2 E_y}{\partial x^2} = \left({k_0}^2 {n_1}^2 - \beta^2\right) E_y & (-\mathrm{a} \le \mathrm{x} \le \mathrm{a}) \\ \dfrac{\partial^2 E_y}{\partial x^2} = \left(\beta^2 - {k_0}^2 {n_s}^2\right) E_y & (\mathrm{x} < \mathrm{a}) \end{cases}$$

Introducing, the wavenumbers κ, σ, ξ, which are the transverse propagation constants:

$$\begin{aligned} \kappa^2 &= {k_0}^2 {n_1}^2 - \beta^2 \\ \sigma^2 &= \beta^2 - {k_0}^2 {n_0}^2 \\ \xi^2 &= \beta^2 - {k_0}^2 {n_s}^2 \end{aligned} \qquad (1)$$

The equations become

$$\begin{cases} \dfrac{\partial^2 E_y}{\partial x^2} = \sigma^2 E_y & (\mathrm{x} > \mathrm{a}) \\ \dfrac{\partial^2 E_y}{\partial x^2} = \kappa^2 E_y & (-\mathrm{a} \le \mathrm{x} \le \mathrm{a}) \\ \dfrac{\partial^2 E_y}{\partial x^2} = \xi^2 E_y & (\mathrm{x} < \mathrm{a}) \end{cases}$$

Now, considering only the case where $n_e = \beta/k$ is higher than n_s and n_0 and lower than n_1, thus $\sigma^2 < 0$, $\xi^2 < 0$ and $\kappa^2 > 0$, and taking into account the boundary conditions (the tangential components E_y and H_z must be continuous at the layer interfaces) the electric field distribution is described by[8–11]:

$$E_y = \begin{cases} \mathrm{A}\ \cos(\kappa a - \phi)\, e^{-\sigma(x-a)} & (\mathrm{x} > \mathrm{a}) \\ \mathrm{A}\ \cos(\kappa x - \phi) & (-\mathrm{a} \le \mathrm{x} \le \mathrm{a}) \\ \mathrm{A}\ \cos(\kappa a + \phi)\, e^{-\xi(x+a)} & (\mathrm{x} < \mathrm{a}) \end{cases} \qquad (2)$$

Since also H_z has to be continuous and $\frac{1}{j\mu_0\omega}\frac{\partial E_y}{\partial x} = H_z$, the continuity of $\frac{\partial E_y}{\partial x}$ leads to the eigenvalues equations

$$E_y = \begin{cases} \kappa \sin(\kappa a + \phi) = \xi \cos(\kappa a + \phi) \\ \sigma \cos(\kappa a - \phi) = \kappa \sin(\kappa a - \phi) \end{cases}$$

$\begin{cases} \tan(u + \phi) = \frac{w}{u} \\ \tan(u - \phi) = \frac{w'}{u} \end{cases}$ where $\begin{cases} u = \kappa a \\ w = \xi a \\ w' = \sigma a \end{cases}$ and

$$u^2 + w^2 = \kappa^2 a^2 (n_1{}^2 - n_s{}^2) \equiv v^2 \quad (3)$$

Thus, we obtain the dispersion relationship

$$\begin{cases} u = \frac{m\pi}{2} + \frac{1}{2}\tan^{-1}\left(\frac{w}{u}\right) + \frac{1}{2}\tan^{-1}\left(\frac{w'}{u}\right) \\ \phi = \frac{m\pi}{2} + \frac{1}{2}\tan^{-1}\left(\frac{w}{u}\right) - \frac{1}{2}\tan^{-1}\left(\frac{w'}{u}\right) \end{cases} \quad \text{for } m = 0, 1, 2, \ldots$$

Defining, $\gamma = (n_s{}^2 - n_0{}^2)/(n_1{}^2 - n_s{}^2)$ (a measure of the waveguide asymmetry) and $b = (n_e{}^2 - n_s{}^2)/(n_1{}^2 - n_s{}^2)$ (the normalized propagation constant), the dispersion equation can be rewritten as

$$2v\sqrt{1-b} = m\pi + \tan^{-1}\sqrt{\frac{b}{1-b}} + \tan^{-1}\sqrt{\frac{b+\gamma}{1-b}} \quad (4)$$

In our simulations the v number were computed using equation (3) while the b number finding the roots of the dispersion equation.

The total effective width of the fundamental mode can be easily computed as

$$h_e = 2a + \frac{1}{\sigma} + \frac{1}{\xi}$$

Where the penetration depth σ inside the top cladding is derived from equation (1)

$$\sigma = \sqrt{k_0{}^2 n_e{}^2 - k_0{}^2 n_0{}^2} = \frac{2\pi}{\lambda}\sqrt{n_e{}^2 - n_0{}^2}$$

With this method, we found that for our core thickness (150 nm) the penetration depth of the evanescent field in the sample media (*X* direction) is roughly 85 nm.

Decreasing the waveguide input taper width (blue line in the Supplementary Fig. 1c-f) significantly increases the effective mode width of the fundamental mode (purple continuous line in Supplementary Fig. 1) leading to a better match with the input beam profile.

Supplementary Fig. 1g shows that the single mode conditions is ensured in both the *X* and *Y* directions: the *b* and *v* numbers (black star) for the *X* configuration (where the asymmetry parameter gamma is equal to 0.1287) and the *b* and *v* numbers (black diamond) for the *Y* direction (where the asymmetry parameter gamma is equal to zero) limits the possible waveguide mode to the fundamental one.

For these simulations, the input beam radius (the distance after which the intensity of the electrical field profile drop of 1/e) has been estimated through the formula [1] (Chapter 2; page 70) and [8](Chapter 11; page 275):

$$w_B = \frac{2\lambda}{\pi}\frac{f_{obj}}{D} \sim 0.23\ \mu m$$

Where D is the laser beam radius, after the beam expander at the back focal aperture of the objective ($D{\sim}6.5mm$), $f_{obj} = 4$ is the focal point of the coupling objective and $\lambda = 647nm$ the wavelength of the beam. This value is comparable with RMS of the Gaussian approximation to the Airy disk[12]:

$$w_B = 0.21\frac{\lambda}{NA} \sim 0.25\ \mu m$$

Coupling efficiency estimation

The first approximate guess of coupling efficiency was estimated based on [8](Chapter 11; page 274-277). The two main contributions to coupling losses are attributed to the waveguide mode field and input beam field distributions mismatch and to the Fresnel reflection.

The efficiency of the filed mode distribution can be approximated through the formula:

$$\eta_W = 4\frac{{w_B}^2{w_M}^2}{({w_B}^2 + {w_M}^2)^2}$$

where w_M is the effective width of the fundamental mode of the waveguide and w_B is the previously defined beam radius.

The efficiency of the transmission can be calculated via

$$\eta_R = 1 - \frac{(n_e - 1)^2}{(n_e + 1)^2}$$

The final total coupling efficiency is thus:

$$\eta = \eta_W * \eta_R$$

In order to compute the effective index of the waveguide at the tip I used the effective index method, which reduces the two-dimensional wave equation into two one-dimensional problems[13]. I assumed my channel waveguide as a rib waveguide (see Supplementary Fig. 3e Right) with an infinitesimal thickness t (5 nm) and a core thickness *h* equal to 150nm while the input tip width w_s has been swept between 0.2 µm up to 1.6 µm. This approximation is not precise for high etching step (large rib height) and small width but it is better than a 1D slab approximation[11,14] and provides the first rough parameters range for finite difference time domain (FDTD) simulations with Lumerical.

The waveguide is approximated with 3 slab waveguides as shown in Supplementary Fig. 3e Right. First the effective index of the Slab 1 and 2 is independently computed along the *X* direction. Secondly, to estimate the confinement in the *Y* direction, a further symmetric waveguide is constructed with a top and bottom cladding with refractive indexes corresponding to the effective index of the Slab 1 waveguides, a core layer with a reflective index corresponding to the effective index of the Slab 2 and a core thickness corresponding to the width of the Slab 2 waveguide. The final analytical solution of this last waveguide provides the effective index of the waveguide.

Figure 5 shows the first approximated results we obtained. The losses due to reflection increase with increasing the taper input width w_s (see Supplementary Fig. 3a Left and 3b; see Fig. 1b for definition of w_s) and the effective width of the mode approach the beam size when w_s is around 150 nm (Supplementary Fig. 3a Right and 3c). This results in a maximal coupling efficiency around 150 nm (Supplementary Fig. 3d).

The chips design can be easily adapted to specific experimental needs, and their fabrication outsourced to commercial companies, such as Ligentec, for direct chip realization and purchase.

Supplementary Note 2: set up the Waveguide platform

Preliminary Step: Chip and holder cleaning [Time: 12 minutes]

Remove sample residual using 2% Hellmanex for 10' at 50°C. Remove further dust with isopropanol. Dry under air flow. Clean the holder with ethanol 30%. Dry with air.

Step 1: Stages positioning and chip-holder mounting. [Time: 13 minutes first time – 3 minutes the following times]

Align the coupling input beam direction with the vertical axis of the imaging column (up-right microscope). Position the X-Y stage at the center of its range. Position the X-Y-Z stage at the center of its range. Align the center of the horizontal stage system with the input beam direction. Place the chip on the positioning slot of the holder. First place the PDMS strip then the gate. Fix the gate. Mount the chip-holder system on the X-Y-Z stage (Supplementary Fig. 8).

Step 2: Waveguide rough coupling. [Time: 3 minutes]

! CAUTION: Use proper safety goggles

Set the laser input power to 5mW. Move the chip position in order to have the input beam at the top edge of the chip. Move the chip along the Y direction (see Fig. 1b) till a weak coupling is establish on the first waveguide. You'll see the light propagation along the waveguide. Move the chip in the Z direction in order to find the position of the chip input facet at the focus plane of the input beam. Use the speckle patter of scatter light, produced by the input beam incident on the input facet, to focus in Z (see Fig. 1b for axis orientation).

Step 3: Focusing of the imaging objective. [Time: 10 minutes first time. 3 minutes following time]

Set the camera exposure time at 100 ms. Remove all the emission filters. Use the waveguide edges as reference to find the focus.

Step 4: Coupling optimization. [3 minutes]

Adjust the waveguide position in order to maximize the scatter light from the top surface of the waveguide.

Supplementary Note 3: Prospective

A multi-well waveguide chip as shown in Supplementary Fig. 18, could be used as a screening platform for the parallel high-throughput imaging of different conditions. More specifically, a DNA-origami nanostructure[15] could be placed in a validation-calibration well independent from the sample well to perform quantitative DNA-PAINT (qPAINT) in a single step.

However, the current wells depth (Supplementary Fig. 18) has to be increased from 2 µm up to 200 µm in order to create a well volume suitable for nanoinjection greater than few nanolitre. Since 200 µm top cladding thickness is not achievable with thin layer SiO_2 deposition, the chip design and fabrication needs to be extended with an additional step involving the spin coating or layer transferring of a thick film such as poly(dimethylsiloxane-block-methyl methacrylate) PDMS.

SUPPLEMENTARY REFERENCES

1. Verdeyen, J. T., Thomas, J. & Verdeyen, J. T. *Laser Electronics*. (1989).
2. Diekmann, R. *et al.* Chip-based wide field-of-view nanoscopy. *Nat. Photonics* 1–9 (2017). doi:10.1038/nphoton.2017.55
3. Thompson, R. E., Larson, D. R. & Webb, W. W. Precise Nanometer Localization Analysis for Individual Fluorescent Probes. *Biophys. J.* **82,** 2775–2783 (2002).
4. Geissbuehler, S. *et al.* Live-cell multiplane three-dimensional super-resolution optical fluctuation imaging. *Nat. Commun.* **5,** 1–7 (2014).
5. Li, Y. *et al.* Real-time 3D single-molecule localization using experimental point spread functions. *Nat. Methods* **15,** 367–369 (2018).
6. Ovesny, M., Krizek, P., Borkovec, J., Svindrych, Z. & Hagen, G. M. ThunderSTORM: a comprehensive ImageJ plug-in for PALM and STORM data analysis and super-resolution imaging. *Bioinformatics* **30,** 2389–2390 (2014).
7. Tinguely, J.-C., Helle, Ø. I. & Ahluwalia, B. S. Silicon nitride waveguide platform for fluorescence microscopy of living cells. *Opt. Express* **25,** 27678 (2017).
8. Clifford R. Pollock and Michal Lipson. *Integrated Photonics*. (Kluwer Academic Publishers, 2003). doi:10.1007/978-1-4757-5522-0
9. Dutta, A., Deka, B. & Pratim Sahu, P. *Planar Waveguide Optical Sensors*. (2016). doi:10.1007/978-3-319-35140-7
10. Okamoto, K. in (ed. Okamoto, K. B. T.-F. of O. W. (Second E.) 1–12 (Academic Press, 2006). doi:https://doi.org/10.1016/B978-012525096-2/50002-7
11. Okamoto, K. in (ed. Okamoto, K. B. T.-F. of O. W. (Second E.) 13–55 (Academic Press, 2006). doi:https://doi.org/10.1016/B978-012525096-2/50003-9

12. Zhang, B., Zerubia, J. & Olivo-Marin, J.-C. Gaussian approximations of fluorescence microscope point-spread function models. *Appl. Opt.* **46,** 1819–1829 (2007).

13. Tuma, M. L. & Beheim, G. Calculated coupling efficiency between an elliptical-core optical fiber and a silicon oxynitride rib waveguide. *NASA Tech. Memo. 106850 h* (1995). doi:10.1117/12.209022

14. Yeh, C. *et al.* Single-mode optical waveguides. *Appl. Opt.* **18,** 1490–1504 (1979).

15. Schmied, J. J. *et al.* DNA origami-based standards for quantitative fluorescence microscopy. *Nat. Protoc.* **9,** 1367–1391 (2014).
