# Supplementary material for: Waveguide-PAINT offers an open platform for large field-of-view super-resolution imaging
Source: Nat Commun. 2019 Mar 20;10:1267. doi: 10.1038/s41467-019-09247-1 (PMC6427008; doi:10.1038/s41467-019-09247-1)
Supplement: Supplementary file 3 — Reporting Summary [file 41467_2019_9247_MOESM3_ESM.pdf]

nature research

Corresponding author(s): S. Manley

Last updated by author(s): Feb 13, 2019

Reporting Summary

Nature Research wishes to improve the reproducibility of the work that we publish. This form provides structure for consistency and transparency in reporting. For further information on Nature Research policies, see Authors & Referees and the Editorial Policy Checklist.

Statistics

For all statistical analyses, confirm that the following items are present in the figure legend, table legend, main text, or Methods section.

n/a	Confirmed	
☐	☒	The exact sample size (*n*) for each experimental group/condition, given as a discrete number and unit of measurement
☐	☒	A statement on whether measurements were taken from distinct samples or whether the same sample was measured repeatedly
☒	☐	The statistical test(s) used AND whether they are one- or two-sided<br>*Only common tests should be described solely by name; describe more complex techniques in the Methods section.*
☒	☐	A description of all covariates tested
☒	☐	A description of any assumptions or corrections, such as tests of normality and adjustment for multiple comparisons
☐	☒	A full description of the statistical parameters including central tendency (e.g. means) or other basic estimates (e.g. regression coefficient) AND variation (e.g. standard deviation) or associated estimates of uncertainty (e.g. confidence intervals)
☒	☐	For null hypothesis testing, the test statistic (e.g. *F*, *t*, *r*) with confidence intervals, effect sizes, degrees of freedom and *P* value noted<br>*Give P values as exact values whenever suitable.*
☒	☐	For Bayesian analysis, information on the choice of priors and Markov chain Monte Carlo settings
☒	☐	For hierarchical and complex designs, identification of the appropriate level for tests and full reporting of outcomes
☒	☐	Estimates of effect sizes (e.g. Cohen's *d*, Pearson's *r*), indicating how they were calculated

Our web collection on statistics for biologists contains articles on many of the points above.

Software and code

Policy information about availability of computer code

Data collection	Microscopy data was collected using the freely available software Micromanager 2.0 beta. All localization data was analyzed using MATLAB 2016b for Windows 10 or the freely available software ThunderStorm.
Data analysis	All data analysis was developed, performed and tested in MATLAB 2016b for Windows 10. All developed code is provided via GitHub. Link: https://github.com/LEB-EPFL/WGmode

For manuscripts utilizing custom algorithms or software that are central to the research but not yet described in published literature, software must be made available to editors/reviewers. We strongly encourage code deposition in a community repository (e.g. GitHub). See the Nature Research guidelines for submitting code & software for further information.

Data

Policy information about availability of data

All manuscripts must include a data availability statement. This statement should provide the following information, where applicable:

- Accession codes, unique identifiers, or web links for publicly available datasets
- A list of figures that have associated raw data
- A description of any restrictions on data availability

The technical drawings and sample data sets can be downloaded from Zenodo https://zenodo.org/record/1475055#.XBkhFFxKhdo. The remaining raw data from this study are available from the corresponding authors upon request.

Field-specific reporting

Please select the one below that is the best fit for your research. If you are not sure, read the appropriate sections before making your selection.

☒ Life sciences ☐ Behavioural & social sciences ☐ Ecological, evolutionary & environmental sciences

For a reference copy of the document with all sections, see nature.com/documents/nr-reporting-summary-flat.pdf

Life sciences study design

All studies must disclose on these points even when the disclosure is negative.

Sample size	Sample sizes were chosen based on how many images could be collected on a given day. Each sample was analyzed separately.
Data exclusions	Datasets with antibodies that stained in a manner inconsistent with the published literature was excluded from the analysis. Data were filtered for quality as described using common metrics in the field (number of localizations, size). Exclusion criteria were not pre-established.
Replication	Multiple replicates were performed with each antibody staining. Staining was variable, and sometimes did not yield data that met the criteria described in "data exclusions."
Randomization	This is not relevant to our study. There was not a group allocation component to the study.
Blinding	There was not a group allocation component to the study.

Reporting for specific materials, systems and methods

We require information from authors about some types of materials, experimental systems and methods used in many studies. Here, indicate whether each material, system or method listed is relevant to your study. If you are not sure if a list item applies to your research, read the appropriate section before selecting a response.

Materials & experimental systems

n/a	Involved in the study	
☐	☒	Antibodies
☐	☒	Eukaryotic cell lines
☒	☐	Palaeontology
☒	☐	Animals and other organisms
☒	☐	Human research participants
☒	☐	Clinical data

Methods

n/a	Involved in the study	
☒	☐	ChIP-seq
☒	☐	Flow cytometry
☒	☐	MRI-based neuroimaging

Antibodies

Antibodies used	All used antibodies are summarized in Methods (Sample Preparation paragraph). Primary antibodies (B512, monoclonal produced on mouse, Sigma T6074) and secondary antibodies PBST are commercial and information regarding validation and application can be found in the manufacturers website.
Validation	Information regarding validation and application can be found in the manufacturers website.

Eukaryotic cell lines

Policy information about cell lines

Cell line source(s)	COS7 cells; Source: ATCC (CRL-1651)
Authentication	The cell line used in this study has not been authenticated by the authors.
Mycoplasma contamination	*Confirm that all cell lines tested negative for mycoplasma contamination OR describe the results of the testing for mycoplasma contamination OR declare that the cell lines were not tested for mycoplasma contamination.*
Commonly misidentified lines (See ICLAC register)	*Name any commonly misidentified cell lines used in the study and provide a rationale for their use.*
